# Corrigenda

**Published:** 1949-01

**Authors:** 


**Corrigenda :** p. 7. For "Thirty-sixth" read "Thirty-seventh."
p. 14. First line of Dr. Corner's article : for "July" read "June".
p. 52. Sixth line : for "being" read "bring".
p. 76. Thirteenth line : for "poililocytosis" read "poikilocytosis."

